# A Mobile Patient-Reported Outcome Measure App With Talking Touchscreen: Usability Assessment

**DOI:** 10.2196/11617

**Published:** 2019-09-27

**Authors:** Marlies Welbie, Harriet Wittink, Marjan J Westerman, Ilse Topper, Josca Snoei, Walter L J M Devillé

**Affiliations:** 1 Research Group Lifestyle and Health Research Center Healthy and Sustainable Living Utrecht University of Applied Sciences Utrecht Netherlands; 2 Institute of Health Sciences, Amsterdam Public Health Research Institute Department of Methodology and Statistics VU University Amsterdam Netherlands; 3 Julius Centre for Health Sciences and Primary Care University Medical Centre Utrecht Utrecht Netherlands; 4 Faculty of Social and Behavioral Sciences University of Amsterdam Amsterdam Netherlands; 5 Dutch Centre of Expertise on Health Disparities (Pharos) Utrecht Netherlands

**Keywords:** mHealth, eHealth, surveys and questionnaires, physical therapy specialty, qualitative research

## Abstract

**Background:**

In the past years, a mobile health (mHealth) app called the Dutch Talking Touch Screen Questionnaire (DTTSQ) was developed in The Netherlands. The aim of development was to enable Dutch physical therapy patients to autonomously complete a health-related questionnaire regardless of their level of literacy and digital skills.

**Objective:**

The aim of this study was to evaluate the usability (defined as the effectiveness, efficiency, and satisfaction) of the prototype of the DTTSQ for Dutch physical therapy patients with diverse levels of experience in using mobile technology.

**Methods:**

The qualitative Three-Step Test-Interview method, including both think-aloud and retrospective probing techniques, was used to gain insight into the usability of the DTTSQ. A total of 24 physical therapy patients were included. The interview data were analyzed using a thematic content analysis approach aimed at analyzing the accuracy and completeness with which participants completed the questionnaire (effectiveness), the time it took the participants to complete the questionnaire (efficiency), and the extent to which the participants were satisfied with the ease of use of the questionnaire (satisfaction). The problems encountered by the participants in this study were given a severity rating that was used to provide a rough estimate of the need for additional usability efforts.

**Results:**

All participants within this study were very satisfied with the ease of use of the DTTSQ. Overall, 9 participants stated that the usability of the app exceeded their expectations. The group of 4 average-/high-experienced participants encountered only 1 problem in total, whereas the 11 little-experienced participants encountered an average of 2 problems per person and the 9 inexperienced participants an average of 3 problems per person. A total of 13 different kind of problems were found during this study. Of these problems, 4 need to be addressed before the DTTSQ will be released because they have the potential to negatively influence future usage of the tool. The other 9 problems were less likely to influence future usage of the tool substantially.

**Conclusions:**

The usability of the DTTSQ needs to be improved before it can be released. No problems were found with satisfaction or efficiency during the usability test. The effectiveness needs to be improved by (1) making it easier to navigate through screens without the possibility of accidentally skipping one, (2) enabling the possibility to insert an answer by tapping on the text underneath a photograph instead of just touching the photograph itself, and (3) making it easier to correct wrong answers. This study shows the importance of including less skilled participants in a usability study when striving for inclusive design and the importance of measuring not just satisfaction but also efficiency and effectiveness during such studies.

## Introduction

### Digital Divide

Electronic health (eHealth) is developing rapidly [1]. It is defined as the use of information and communication technology (ICT) in health care [2]. A growing amount of literature indicates that using eHealth can improve the accessibility, quality, and efficiency of health care [3-5]. It seems to be effective for people who have access to it and are able to use it well, which is not the case for everybody [6,7]. For instance, people with low income or low education and people who are 65 years and older are vulnerable when it comes to effective eHealth use. In these populations, access to the internet and hardware, such as personal computers, tablets, mobile phones, and smartphones, and the experience and skills to use these devices is low [6-9]. Differences between people regarding digital skills and access to the internet and hardware is often referred to as the digital divide [10,11]. As eHealth technologies are usually primarily developed for people who are experienced and skilled in using ICT [12,13], people who do not have access to ICT or are not skilled in using it are at risk of being excluded from the use of eHealth. Looking at the widespread expansion of eHealth technologies, this encompasses the potential threat of contributing to the ongoing exacerbation of health inequalities in Western countries [1]. However, if the needs, preferences, capacities, values, and goals of potential users who do not have good access to the internet and digital technology or who are not well skilled in using this technology would be explored and taken into account during each stage of development of eHealth tools, eHealth could potentially *reduce* health inequalities [14].

### Mobile Technology Reduces Digital Divide

The development of a specific form of eHealth technology, called mobile health (mHealth) technology, seems especially promising when it comes to reducing health inequalities [5,15-17]. mHealth has been defined by the Global Observatory for eHealth of the World Health Organization as “medical and public health practice supported by mobile devices, such as mobile phones, patient monitoring devices, personal digital assistants, and other wireless devices” [18]. A recent project called eSalud showed that mHealth can be cost-effective, help to overcome cultural and language barriers, and provide health information and services to low–health access areas [15]. Furthermore, recent publications indicate that the digital divide is narrowing because of the increased ownership of mobile devices such as smartphones and tablets [5,16,17].

### Inclusive Mobile Health Design Could Potentially Reduce Health Inequalities

Still, having access to the internet and digital technology does not automatically mean that people are able and willing to use it effectively to increase their health or that different people use it in the same way [14,19-25]. Recent studies found ethnic and socioeconomic differences in mHealth usage [19,20], and it is known that older people use mHealth differently from younger people [14]. In addition, though the gap of people owning tablets and smartphones between groups is closing, still a substantial number of people do not own such devices. For instance, the percentage of Dutch citizens of 65 years and older owning a tablet computer in 2017 was 55.2% versus 75.8% citizens of 12 to 25 years of age [26]. Considering that vulnerable groups, such as people with low income and low education, bear a disproportionate burden of disease [27,28] and the number of health care visits increases with age [29], it is to be expected that a relatively large number of care recipients do not have a lot of experience in using mobile technology. To fulfill the promise of mHealth technology contributing to a reduction of health inequalities, it is very important to carefully test the usability of mHealth apps in research populations, which include members of the target populations that are at risk of being excluded from usage of the tested tool.

### Development of the Dutch Talking Touch Screen Questionnaire

In the past years, a prototype of an mHealth app, called the Dutch Talking Touch Screen Questionnaire (DTTSQ), was developed in The Netherlands. The idea of developing a talking touch screen was inspired by the work of Hahn and Cella [30]. The aim of developing the DTTSQ was to enable Dutch physical therapy patients to autonomously complete a user-friendly health-related questionnaire regardless of their literacy and digital skills. As it is not to be expected that all physical therapy patients own a tablet computer, the DTTSQ is meant to be presented in a physical therapy practice on a tablet computer that is owned by the physical therapy practice concerned. Patients are asked to complete the DTTSQ in the waiting room of the physical therapist before their first visit. The development of the prototype of the DTTSQ, which runs on a tablet computer, was described in detail by Cremers et al in 2015 [31]. Before this study, the prototype was only tested in a sample *outside* of the physical therapy context.

The aim of this study was to test the prototype of the DTTSQ within the physical therapy context to see what parts of the prototype needed adjustment for it to be user-friendly for physical therapy patients regardless of their level of experience with operating mobile technology.

The research question underlying this study was:

What is the usability of the prototype of the DTTSQ for physical therapy patients with different levels of experience in using mobile technology?

## Methods

### Design

A qualitative descriptive study was carried out. Observational data on the way participants operated the DTTSQ were collected through the Three-Step Test-Interview (TSTI) method [32]. This method includes both think-aloud and retrospective probing techniques.

### Definitions

*Usability* was defined by the International Standards Organization as “the effectiveness, efficiency and satisfaction with which specified users can achieve goals in particular environments” [33]. *Effectiveness* is the accuracy and completeness with which users achieve certain goals [34]. In this study, problem rates and severity of problems were used as the primary indicator of effectiveness. *Efficiency* is the relation between the accuracy and completeness with which users achieve certain goals and the resources expended in achieving them [34]. In this study, completion time was used as an indicator of efficiency. *Satisfaction* is the users’ comfort with and positive attitudes toward the use of a system [34]. In this study, participants were interviewed about their satisfaction with the ease of use of the DTTSQ. Ease of use was defined as the degree to which the usage of a particular system is free from effort [35].

### Setting and Participant Selection

Data were collected in the same study population and at the same time as the data reported in a paper earlier published by Welbie et al [36]. Recruitment took place in 11 primary care practices in deprived areas of Utrecht, The Netherlands. Patients were invited by their physical therapists to participate in this study. The physical therapists shortly explained the goal of the study and provided the patients with an information letter that was written in plain Dutch language. If patients were interested, the physical therapist asked permission to give the patients’ telephone number to researcher IT. Then researcher IT (1) contacted the patient by telephone, (2) again shortly explained the aim of the study, (3) made sure the patient understood what was asked of him/her, (4) answered any question the potential participant may have had, and (5) checked the inclusion criteria. The inclusion criteria for participants were as follows: aged 18 years or older, Dutch as their first language, and the patients and both their parents were born in The Netherlands. This last inclusion criterion was added because in a following study, the usability of a direct Turkish translation of the DTTSQ will be tested. For the outcomes of both studies to be comparable, it is important that the cultural background of participants of this study was not *mixed*. This last inclusion criterion excludes second-generation immigrants with a non-Dutch background. The sampling procedure was aimed at getting a broad variation in levels of education and age plus balance in our sample regarding gender. Age was used as a proxy for level of experience with using mobile technology because with increase in age, the experience with mobile devices decreases [26]. Taking age as a selection criterion was more practical for the recruiting physical therapists, as this is noted standardly in patient files. By making sure that there was variation in age, it was expected to find variation in experience with mobile devices in the study sample. Throughout the recruitment process, the recruiting physical therapists were constantly kept informed about the profiles of participants the researchers were looking for. In total, 24 physical therapy patients were included in this study [36]. Characteristics of the study population can be found in Tables 1 and 2.

**Table 1:** Characteristics of study population (N=24).

Characteristics		Study population
Age (years), mean (range)		56 (18-79)
**Gender, n (%)**		
	Male	9 (38)
	Female	15 (62)
**Level of education, n (%)**		
	Low^a^	6 (25)
	Moderate^b^	13 (54)
	High^c^	5 (21)
**Self-declared experience with using mobile technology, n (%)**		
	None	9 (37)
	Little	11 (46)
	Average/high	4 (17)

^a^Low: no or at most primary education finished.

^b^Moderate: lower secondary education, (upper) secondary education, or postsecondary nontertiary education (including vocational education).

^c^High: tertiary education (bachelor’s degree or higher).

**Table 2:** Characteristics per participant.

Pseudonym	Experience with mobile technology	Age (years)	Level of education
Ida	None	66	Low^a^
Bill	None	72	Moderate^b^
Mia	None	73	Moderate
Dora	None	77	Low
Ilene	None	79	Low
Bob	None	68	Moderate
Jerome	None	47	Low
Helga	None	54	High^c^
Michelle	None	56	Low
Roger	Little	70	Moderate
Peter	Little	18	Moderate
Christine	Little	39	Moderate
Jill	Little	55	High
Lydia	Little	56	Moderate
Rose	Little	60	Moderate
Francine	Little	61	Moderate
Harald	Little	63	High
Henry	Little	64	Moderate
Ronald	Little	70	Low
Bernie	Little	76	High
Jude	Average/high	18	Moderate
Joline	Average/high	19	Moderate
Ellen	Average/high	32	High
Sandra	Average/high	39	Moderate

^a^Low: no or at most primary education finished.

^b^Moderate: lower secondary education, (upper) secondary education, or postsecondary nontertiary education (including vocational education).

^c^High: tertiary education (bachelor’s degree or higher).

### Content of the Dutch Talking Touch Screen Questionnaire

The prototype of the DTTSQ was a digital app on a tablet computer. It was developed during a co-design process [37], which in this case meant that a group of 10 low-literate people helped to design the questionnaire. As a result of the co-design process, questions on pain location and pain intensity were added to the original questions of an existing questionnaire which aims to select limitations in functioning and to formulate specific treatment goals [38,39]. Furthermore, visual (videos and photos) and auditory (speech technology) support were added to enable participants to see and hear the questions that were shown on separate screens. Response items could be selected by tapping on the touch screen and plain language was used in all spoken and written text within the DTTSQ [31]. An overview of all types of screens is given in Multimedia Appendix 1. The 8 questions of the questionnaire can be found in screenshots 2, 3, 4, 7, 9, 11, 12, and 13, which can be found in Multimedia Appendix 1.

#### Instructions

**Figure 1:**
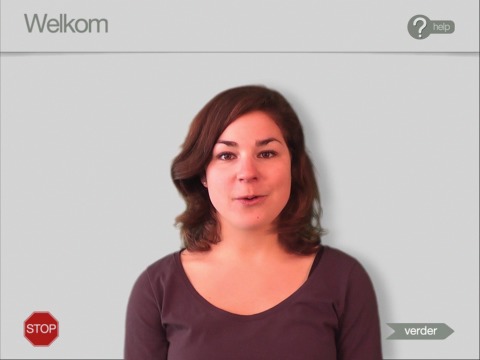
Introduction movie.

Instructions were given in the form of 3 video clips:

1. An introduction clip in which the purpose of the questionnaire and all functions of the questionnaire were explained (see Figure 1 and screenshot 1 in Multimedia Appendix 1).
2. An instruction clip in which the purpose of question 4 and a newly added navigation function were explained (see screenshot 6 in Multimedia Appendix 1).
3. A closing clip in which the participant is thanked, explained what the physical therapist would do next, and told that the questionnaire would close down automatically (see screenshot 16 in Multimedia Appendix 1).

#### Functions

#### Next Button

**Figure 2:**
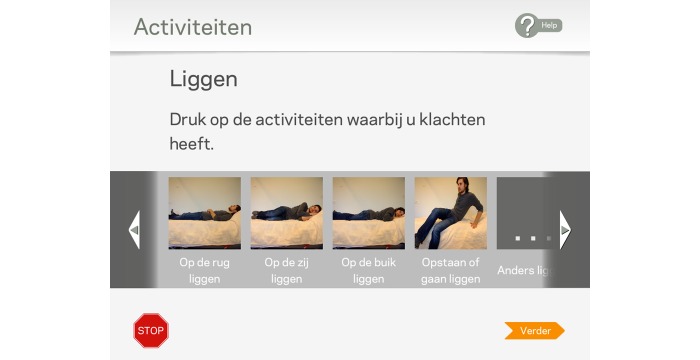
Question 4: “Select the activities in which you are limited”.

It is a navigation function to go to the next screen. It is not activated unless a response item is selected (except for question 4; see Figure 2 and screenshot 7 in Multimedia Appendix 1).

#### Help Button

It activates the help function: the text on the screen is read aloud, the purpose of the question is explained, and operating instructions for the particular screen are provided.

#### Correction Function

Tapping a second time on a response item, deselects the item.

#### Stop Button

It is an escape function: it shuts down the questionnaire. All previous given answers are saved.

#### Overviews

**Figure 3:**
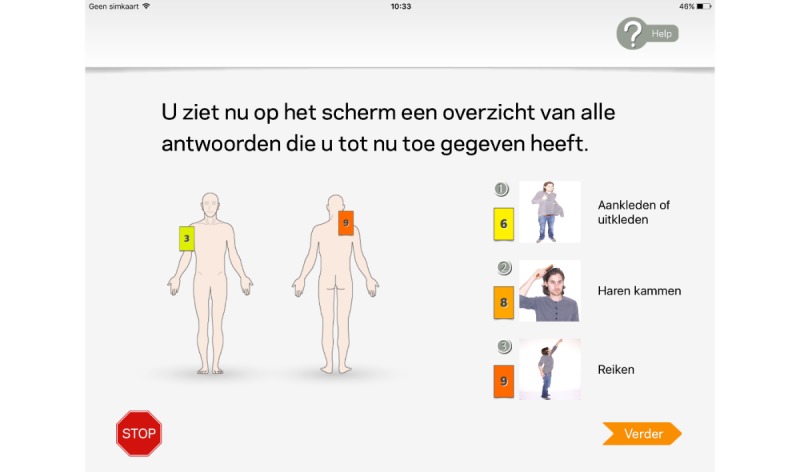
Overview answers total questionnaire: “On the screen you see an overview of all your answers you provided until now.”.

To help participants keep track of their answers, overviews of previous given answers were provided regularly during completion of the questionnaire (see Figure 3 and screenshot 5, 8, 10, 14 and 15 in Multimedia Appendix 1 screenshots).

### Data Collection and Procedures

Data collection took place at the physical therapy practice or the participant’s home, depending on the preference of the participant. Researchers IT and JS were present. Researcher IT was in the lead during the interviews. Researcher JS asked complementary questions if she missed information.

The following steps were taken according to the TSTI method [32].

#### Step 1

Each participant was observed by researchers IT and JS while they were completing the DTTSQ thinking out loud. This step was aimed at collecting observational data regarding the usability of the DTTSQ. The data collected consisted of 2 kinds: (1) observations of participant’s behavior and (2) think-aloud data. The data were recorded on videotapes as well as audiotapes. In addition, the researchers took real-time notes for use during the following steps of the interviews as well as for later analysis. The researchers wrote their notes down on hardcopies of print screens of the DTTSQ. Researchers IT and JS noted problems with operating the tablet computer, including using the touch screen, navigating through the questionnaire, understanding the task given in each screen, selecting response items, and using the correction function. They also wrote down when the stop button was used. The researchers did not interfere in the completion process by asking any questions or providing help.

#### Step 2

Researcher IT conducted an in-depth interview after the participant finished completing the DTTSQ. Data collection during this step was exclusively focused on filling possible gaps and checking the observational data collected during step 1.

#### Step 3

During step 3 of the TSTI, researcher IT conducted a semistructured interview aimed at eliciting experiences and opinions of the participant. During the interview, each screen was operated in the same way the participant did during step 1 and the same answers were entered. This was done to help the participant to clearly remember all his thoughts and actions during the completion of the questionnaire. Participants were stimulated to report feelings and express opinions, preferences, and recommendations. If they encountered problems in operating the DTTSQ, they were asked what they thought the exact nature and possible cause of each type of problem was and how they tried to overcome the problem. Then, the participants were questioned about their satisfaction regarding the ease of use of the user interface, technical operation, layout and content, and overall usability of the DTTSQ. Researcher JS was allowed to ask complementary questions, if she felt it was necessary, to get complete and enriched data. Researcher IT finished the interview by collecting demographic data and data on self-reported experience with mobile technology (see Tables 1 and 2).

### Analyses

Data were analyzed using a thematic content analysis approach [40]. Overall, 4 types of data were analyzed: (1) video recordings of the completion of the questionnaire, (2) field notes of the observed participant behavior, (3) transcriptions of the audio recordings of the semistructured interviews, and (4) background information regarding the educational level, age, gender, and self-reported experience with using mobile technology.

To get more familiar with the data and to create an overview, researcher MW made a descriptive summary of each case on the basis of all 4 types of generated data. Each summary contained information on whether or not the questionnaire was fully completed, if, when, and why the stop function was used, the kind of problems that occurred with the operation, the completion time, and all emerging themes regarding satisfaction or dissatisfaction with the ease of use of the questionnaire. The summaries were supplemented with information regarding educational level, age, gender, and experience in using mobile technology.

Subsequently, researcher MW derived the observed problems from the summaries. She clustered the problems. For every new problem, a new category was made. MW analyzed the video recordings to see how many times each problem was made in total, per participant and per question/screen of the questionnaire. After a full overview of problems had emerged, she scored the level of severity of each problem, as described by Nielsen and Loranger [41]: low, medium, serious, or critical. To score severity, she used the method of Hattink et al [42]. The severity was scored by answering the 3 questions of Nielsen and Loranger [41] with *yes* (=one point) or *no* (=0 points):

1. Frequency: Do a substantial number of users encounter the problem? Within this study, this question was answered with yes if one-third or more participants had encountered the problem.
2. Impact: Does the problem cause much trouble to those users who encounter it? Within this study, this question was answered with yes if the problem had led at least one participant to stop completing the questionnaire.
3. Persistence: Does the problem cause trouble repeatedly? Within this study, this question was answered with yes if the problem had occurred with an average of more than one time per participant.

This resulted in a 0- to 3-point score per problem. Each score was related to a level of severity: 0=low, 1=medium, 2=serious, and 3=critical.

These severity ratings give an indication of which problems lead to disastrous usability problems and which problems are more cosmetic in nature [43]. This provides insight into whether or not the usability of the DTTSQ needs to be improved before it can be released. Nielsen and Loranger recommend tackling only serious and critical severe problems during the development process of a digital tool. Low and medium severe problems do not have priority according to Nielsen and Loranger because although they are bothersome, they are not likely to directly influence the usage of a tool. This makes it uninteresting to tackle them from a cost-benefit perspective. Serious and critical severe problems on the contrary can be so disrupting that they can make users stop using a tool or prevent them from even starting to use it at all. Therefore, they should not be ignored during the development process of a digital tool [41].

As a next step, researcher MW started open coding of all fragments in the transcripts of the semistructured interviews that were related to (dis)satisfaction about the ease of use of the questionnaire using MAXQDA 10 (VERBI Software). After she finished open coding, she organized and structured the codes until a coding scheme emerged on the basis of which the part of the research question that was related to satisfaction of the participants could be answered sufficiently.

As a last step, researcher MW ordered the analyzed data into 3 groups: data of participants who had (1) no, (2) little, and (3) average/high experience in using mobile technology. This was done to see whether or not data differed within and between these groups.

During the whole course of the study procedures, coding, analysis steps, and interpretation decisions were discussed with researchers HW, MJW, and WD.

### Ethics

No external funding was received by the Utrecht University of Applied Sciences to conduct this study. This study was submitted to the medical ethics committee of the Academic Medical Centre of Amsterdam which declared that it does not fall under the scope of the *Medical Research Involving Human Subjects Act*. The study was conducted according to the principles of the Declaration of Helsinki. All participants provided written informed consent. The participants’ names used in this article are all fictitious to protect their privacy.

## Results

### Effectiveness

Overall, 9 out of the 24 participants in this study did not complete the DTTSQ fully (see Table 3). Michelle (56 years), Bill (72 years), and Helga (54 years), who were all inexperienced in using mobile technology stopped completing the questionnaire by using the stop button. Inexperienced Ida (66 years), Ilene (79 years), Dora (77 years), and Mia (73 years) and little-experienced Peter (18 years) and Rose (60 years) went through the whole questionnaire but unintentionally left one or more parts open.

**Table 3:** Experience with mobile technology and completion of the Dutch Talking Touch Screen Questionnaire.

(Sub)Population	Not fully completed	Fully completed
No experience using mobile technology (n=9)	7	2
Little experience using mobile technology (n=11)	2	9
Average/high experience using mobile technology (n=4)	—	4
Total population (N=24)	9	15

### Unanswered (Parts of) Questions

Inexperienced Michelle (56 years), Ida (66 years), Ilene (79 years), Dora (77 years), and Mia (73 years) and little-experienced Peter (18 years) and Rose (60 years) failed to fully complete the DTTSQ because they failed to select answering options and/or unintentionally skipped questions by double-tapping on the next button (see problems 1-5 in Table 4). All participants, except for Michelle, additionally failed to notice that they had not effectively selected an answer because the difference between activated and nonactivated answers was not accentuated enough (see problem 6 in Table 4).

### Use of the Stop Button

When inexperienced Michelle (56 years) noticed most of her answers were missing from the summary in question 6, she got confused. In question 6, she was asked to choose the 3 most important activities in which she was limited. The screen contained only 1 activity photo whereas, in her mind, she had selected a lot of photo’s earlier. Except for the 1 photo that she had managed to select, she had tapped on the text beneath the photos, in which case, the item was not activated (see problem 5 in Table 4). The activity on the 1 photo that she had managed to select was of no priority to her. Therefore, she decided to use the stop button and ended the questionnaire.

Inexperienced Bill (72 years) had a lot of trouble operating the questionnaire. He commented on the introduction clip:

I do not think that what she is saying is difficult, but I just am not able to remember it. I have no experience with these kind of devices. So I forgot what she said right away.

Bill managed to get to question 4 by activating the help function on each screen he entered. When he touched the navigation button to see all the activity photos in question 4, the photo gallery moved in a different direction then he had presumed. This startled him somewhat and made him forget that he had to push the next button to go to the next screen (see problem 7 in Table 4). He activated the help function again, but that was of no use anymore. After trying a few buttons without succeeding to go to the next screen, he gave up and tapped on the stop button.

Inexperienced Helga (54 years) operated the digital questionnaire fluently until she had to choose the 3 activities that were most important to her in question 5. She did not use the navigation function of the photo gallery and as a result she did not see all her earlier selected activities (see problem 4 in Table 4). She chose the 3 most important activities out of the 5 photos that were immediately visible. When she realized what happened, she wanted to pause for a moment to find out how she could change her answer. She interpreted the stop button as a *time-out function* and was a bit shocked when she found out that she had stopped the questionnaire altogether.

A complete overview of frequency and severity of all problems encountered can be found in Table 4.

**Table 4:** Frequency and severity of encountered problems during the completion processes of all participants.

Problem	Number of participants	Frequency	Severity rating
1. Accidently skipping a screen by double tapping on the next button	8	16	Serious
2. Double-tap on answering option causing activation and deactivation of the answer of choice	1	1	Low
3. Skipping a screen by accidently touching the next button with the palm of the hand	1	1	Low
4. Not using the navigation function of the photo gallery in question 4 causing the participant not seeing all presented response items	2	2	Medium
5. Touching the text underneath a photo in question 4 to select an activity instead of touching the photo itself causing the activity not to be selected	3	30	Serious
6. Not able to see whether or not a selected answer is activated (not accentuated enough)	8	8	Medium
7. Not knowing how to get to the next screen	1	1	Medium
8. Pushing too hard or tapping too soft on the touch screen causing the touch screen not to respond	11	40	Serious
9. Not able to correct a wrong answer	8	13	Serious
10. Not reading the text above the photos of question 5 causing the participant to keep on performing the task given with question 4	4	8	Medium
11. Not noticing that the multiple numerical rating scale-effort scores in question 8 are related to different activities, which by mistake results in identical scores for different activities	1	1	Low
12. Scoring the body chart in question 2 mirrored	2	2	Low
13. Scoring (serial) questions that do not apply to the participants’ situation (forced by the software)	1	4	Medium

### Number of Problems

Average-/high-experienced Ellen (32 years), Sandra (39 years), and Joline (19 years) and little-experienced Jill (55 years), Lydia (56 years), and Christine (39 years) were able to complete the questionnaire without any problems. The other 18 participants were not able to operate the questionnaire fluently. In an absolute as well as relative sense, more participants with no experience in using mobile technology encountered problems during the completion of the DTTSQ than little-experienced participants did (see Table 5). Inexperienced participants encountered an average of 3 problems per person, whereas participants with little experience encountered an average of 2 problems per person. Within the subgroup of average-/high-experience participants, only 1 person encountered ` problem during completion (see Table 5). A total of 11 participants encountered problem 8, “Pushing too hard or tapping too soft on the touch screen causing the touch screen not to respond” multiple times (see Table 4). In some case, participants looked startled after problem 8 occurred. In these cases, researcher IT encouraged the participant to go on by kindly saying *try again*.

**Table 5:** Number of participants encountering each problem per level of experience with using mobile technology (N=24).

Problem	No experience (n=9)	Little experience (n=11)	Average/high experience (n=4)	Total population
1	5	3	—^a^	8
2	1	—	—	1
3	—	1	—	1
4	1	1	—	2
5	2	1	—	3
6	4	4	—	8
7	1	—	—	1
8	6	5	—	11
9	3	4	1	8
10	2	2	—	4
11	1	—	—	1
12	1	1	—	2
13	—	1	—	1

^a^Not applicable.

### Efficiency

The 21 participants who got to the end of the questionnaire had an average completion time of 10 min and 25 seconds. Inexperienced participants needed more time than little-experienced participants did, who in their turn needed more time than average-/high-experienced participants did (see Table 6).

**Table 6:** Completion time of all participants who did not end the questionnaire prematurely.

(Sub)Population	Mean completion time (min)	Median completion time (min)	Range of completion times (min)
No experience with mobile technology (n=6)	11.38	9.38	8.2 to 22.10
Little experience with mobile technology (n=11)	10.41	9.57	6.54 to 18.10
Average/high experience with mobile technology (n=4)	7. 55	7.42	5.50 to 10.26
Total population (n=21)	10.25	9.43	5.50 to 22.10

### Satisfaction

All participants were satisfied with the ease of use of the questionnaire. The use of plain language, the way ICT was used, and the way the user interface was designed were greatly appreciated by the participants:

Everything was well described. I am not always able to understand everything, but this went well. I understood what was asked of me.

Inexperienced Dora, 77 years

I have trouble operating my mobile phone and I own a notebook but don’t you ask me how that thing works! I am capable of a lot but I am not technical in that way. [...] This was the first time for me to use a tablet computer. I only had to follow the instructions. I did not have to start it up or open something, it just started working and it shut down by itself. I thought it was easy to work with. Better than when you have to write things down.

Little-experienced Roger, 70 years

I am a very visual person. And this thing is very visual. [...] Like green is ‘no pain’ and red is ‘a lot of pain'.

Average/high experienced Ellen, 32 years

All participants were satisfied with the completion time of the DTTSQ.

### Satisfied Despite Encountering Problems

Operation problems, regardless of the amount and severity of the problems encountered by each individual participant, did not influence satisfaction about the ease of use of the questionnaire. Little-experienced Francine (61 years), for instance, was asked how she felt about the fact that the app did not always respond to her touch right away (see problem 8 in Table 4). She encountered this problem 13 times in total. She lightheartedly answered as follows:

*Oh these are things that happen. I experience the same things with my own computer. My computer refuses to sometimes, so... I think I was just pushing too hard on the tablet sometimes, that’s all.*

When inexperienced Bill (72 years), who used the stop button, was asked if he would have preferred a paper-based questionnaire he said the following:

*No. It took me some time to get used to it but it is easy to use actually.*

### Expectations Exceeded

A total of 9 participants explicitly stated that operating the questionnaire was easier than they had expected beforehand. When inexperienced Ida (66 years) was confronted with the questionnaire she agitatedly said the following:

*Never in a million years I believe I can do this. That I can tell you right away.*

Noticeably reluctant and nervous, she started to complete the questionnaire. When she finished, she seemed surprised and relieved. She smiled and said the following:

*Okay? So this was the questionnaire? [...] Ooooh but this was doable! I thought I would have to look up things and operate it like my grandchildren do.*

And then she started laughing out loud and cheerfully asked if anyone would like to have some coffee.

Little-experienced Christine (39 years) was positively surprised too:

*It responds really well. Normally I am not that good with screens, but this is easy. It almost feels like a game! It really responds nicely. Nothing disappears when I touch it. It reacts very calmly but at the same time it is very fast. I really like that it contains photo’s instead of drawings. It is instantly clear: these are my activities and that is what they mean by “sitting down”. You see it right away. I also like the regular summaries. It keeps you on track and enables you to check whether or not you forgot something.*

### Participants’ Recommendations for Improvement

The most mentioned recommendations for improvement of the usability of the DTTSQ by participants were: shorten the length of the instructions, accentuate the activated response items, and improve the user interface of question 4 by giving participants a complete overview of activities to choose from in one screen, without having to use complicated navigation functions.

## Discussion

### Principal Findings

All participants within this study were very satisfied with the ease of use of the DTTSQ. Overall, 9 participants stated that the usability of the app exceeded their expectations. The participants who had no experience with using mobile technology completed the prototype of the DTTSQ less effectively and efficiently than the little- and average-/high-experienced participants did. In the group of average-/high-experienced participants, only 1 problem was encountered in total, whereas the inexperienced participants encountered an average of 3 and the little-experienced participants an average of 2 problems per person. Overall, 13 different kind of problems were encountered during this study. From a cost-benefit perspective, 4 of these problems will need to be addressed during future development of the DTTSQ because they have the potential to influence the future usage of the tool negatively [41]. The 4 problems that need to be addressed are problem 1 “Accidently skipping a screen by double tapping on the next button,” problem 5 “Touching the text underneath a photo in question 4 to select an activity instead of touching the photo itself causing the activity not to be selected,” problem 8 “Pushing too hard or tapping too soft on the touch screen causing the touch screen not to respond,” and problem 9 “Not able to correct a wrong answer.” Participants also recommended to shorten the length of the instructions and improve the user interface of question 4 by giving participants a complete overview of activities to choose from in one screen, without having to use complicated navigation functions.

### Comparison With Previous Work

In earlier studies, talking touch screens were found to be easy to use for people with different levels of education, literacy, or digital skills. These conclusions were based on study participants’ level of satisfaction with the ease of use of the tool [44,45] or on results on satisfaction combined with the efficiency with which the tool was completed [46-50]. Effectiveness was not, or in case of Vargas et al very slightly [45], tested. This is a debatable approach, because Frokjaer et al consider effectiveness, efficiency, and satisfaction as independent aspects of usability and state that it is risky to assume that there are correlations between these aspects [34]. Therefore, according to Frokjaer et al, satisfaction and efficiency outcomes should always be tested in combination with outcomes of effectiveness to give a complete and realistic overview of the usability of a tool. The results of this study confirm the necessity of combining all 3 aspects of usability during usability studies. All participants in this study, including participants who were not able to fully complete the questionnaire because of problems they had with operating the app, were satisfied with the usability of the DTTSQ. Looking solely at the results on satisfaction with the ease of use (which were also found in the comparable studies [44-50]) one could make the assumption that the DTTSQ is, usability wise, ready to be released. Looking at the data found on efficiency within this study, one can see that more-experienced participants need less time to complete the questionnaire. This seems logical and matches the results of comparable studies [46,49]. In addition, the completion time was acceptable to all participants of this study. On the basis of the efficiency results solely, one could also conclude that the DTTSQ was ready to be released. Looking at the results on effectiveness and specifically at the severity rates of the problems that occurred during the response process though, the researchers of this study concluded that the usability of the DTTSQ needs to be improved to prevent problem 1, 5, 8, and 9 from occurring before it can be released.

The results of this study show how difficult it is to strive for an *inclusive design*. A lot of effort was put into developing a tool that is easy to use for potential users at risk of exclusion from usage of mHealth tools [31]. By choosing a co-design strategy, development of a user-friendly tool for people with diverse levels of education, literacy, and digital skills was taken a step further than what was done in earlier comparable projects [44-50]. In the other projects, users were involved in the evaluation process of the tools, but development was done by designers and health professionals. In spite of the user-centered development approach that was taken during the development process of the DTTSQ, the goal of inclusive design was not reached yet. Looking at the results of this study, the tool is ready to be released for average-/high-experienced users but not for less-experienced future users. To be able to evaluate the worth of including potential users at risk of exclusion, it would be interesting to be able to compare data on efficiency and effectiveness of talking touch screens that were developed earlier. Specifically, because the user interface and structure of the DTTSQ differs from comparable tools. For instance, the screen of the DTTSQ contains fewer buttons and operation functions, it does not have a back function, it provides summaries of given answers regularly to the respondent, and questions are not automatically read out loud. In addition, the design and format of the answering options in the earlier developed talking touch screen [44-50] does not match the recommendations given by the low-literate people that helped to design the DTTSQ [31]. If it would be possible to compare results on effectiveness from the tests of several different kind of talking touch screens, a lot of insight could be gained in what does and does not work in striving for an inclusive design for less-skilled users of such tools.

According to Frokjaer et al, relations between the 3 aspects of usability depend in complex ways on the app domain, use context, and user’s experience [34]. User’s experience may well have been of influence on the satisfaction outcomes of this study. Overall, 83% (20/24) of the total study population had no or little experience in using mobile technology (see Tables 1 and 2). Limited or no user experience may have caused a form of computer anxiety, resulting in low self-efficacy, which in turn led to low expectations toward the ease of use of the DTTSQ [51]. A total of 9 out of the 24 participants in this study explicitly stated that operating the DTTSQ was easier than they had expected beforehand. The other participants did not explicitly state this, but their statements on the ease of use could easily be interpreted as such. No participant stated or gave the impression that the ease of use of the DTTSQ was lower than they would have expected. According to the Expectation Confirmation Theory [52], actual performance exceeding the expectations of testers leads to satisfaction among these testers. The more their expectations are exceeded, the more satisfied testers will become. Owing to the limited user experience of most of the study participants, expectations toward the ease of use of the DTTSQ may have been low, which may have made it easier to exceed them. Especially considering that the DTTSQ was specifically designed to be easy to use for low-educated people who lack the necessary skills to use ICT [31]. Looking at the results of studies that evaluated the satisfaction about the ease of use of earlier developed talking touch screens, a similar picture of highly satisfied study participants emerges [44-50]. The qualitative results in 2 of these studies also show that participants’ expectations regarding the ease of use of the tested tool were exceeded [44,47] and 2 other authors report that satisfaction among the study participants was *extremely* and *overwhelmingly* high [45,48]. In all of the comparable studies, a large proportion of the study participants had no or limited computer experience [44-50]. It is reasonable to assume that limited computer experience may have led to low expectations regarding the ease of use of the talking touch screens and, therefore, played a role in the high satisfaction outcomes.

### Strengths and Limitations

It is a strength of this study that all 3 aspects of usability, instead of just satisfaction and efficiency, were thoroughly tested and that all of the results of the tests were differentiated for inexperienced and little- and average-/high-experienced users (which was not the case in the reports of the comparable studies [44-50]). To this date, this is the first study on usability of talking touch screens that has taken this approach. As a result, an insight was gained into what kind and amount of usability problems are encountered by the most vulnerable group of potential users.

It is a strength in itself that inexperienced as well as little and average-/high-experienced users of mobile technology were included in this study. Although recommended in the literature [12,53], to this date, there has been an insufficient number of empirical studies to prove the worth of involving future users at risk of exclusion in the development process of eHealth tools [54]. In a recent review, Latulippe et al found only 3 studies that involved future users at risk of exclusion in their design and evaluation processes [8]. This study contributes to the body of knowledge of inclusive mHealth design which involves active participation of vulnerable potential users in usability evaluation.

The qualitative TSTI method [32] was chosen for data collection in this study. This method was never used in a usability study before. The results of this study show that the TSTI method is suitable to gain insight into the usability of mHealth tools. It helped the researchers to understand not only what kind of usability problems occurred but also what caused these problems to occur and what effect encountering the problems had on participants. In addition, this method suited the needs of low-educated and low-literate participants by not demanding any reading or writing skills from them. A downside of the chosen method is the lack of generalizability of the data.

A limitation of this study was that participants were encouraged by the interviewer to try touching the screen again when they looked startled because it did not react to their initial touch. This may have influenced the results on effectiveness because it is unknown what would have happened if the interviewer would not have interfered. This may vary from no effect because the participant would have tried it again anyway, to a higher frequency of occurrence of problem 8, to more participants prematurely stopping to complete the DTTSQ because of being under the impression that the app had stopped working. Any kind of interference in the process of usability testing has a direct influence on the effectiveness results and possibly also on the efficiency and satisfaction results and should therefore be avoided.

### Conclusions

The usability of the DTTSQ needs to be improved before it can be released. No problems were found with satisfaction or efficiency during the usability test. Effectiveness needs to be enhanced by (1) making it easier to navigate through screens without the possibility of accidently skipping one, (2) enabling the possibility to insert an answer by tapping on the text underneath a photograph instead of just touching the photograph itself, and (3) making it easier to correct wrong answers. Participants additionally recommended to minimize the length of the instructions and present all the answering options of question 4 in one screen.

### Directions for Future Research

During further development of the DTTSQ, both the results of this study and the study on response process of the DTTSQ [36] should be taken into account simultaneously. The usability and the response processes will have to be retested in exactly the same manner after adjustments in the DTTSQ have been made. This process will have to be repeated until an acceptable level of usability and face validity of the DTTSQ are reached. The next step in research should be quantitative usability, validity, and reliability testing producing generalizable data.

Considering the difference in the number of problems encountered by inexperienced and little-experienced participants versus average-/high-experienced participants within this study, it can be concluded that in striving for an inclusive design, it is vital to involve potential users at risk of exclusion during further development and testing of the DTTSQ. Selecting quantitative methods for this purpose may be quite challenging because the researchers will have to develop a quantitative study design that will enable people with low literacy skills and low educational levels to participate. Research designs that include reading and writing tasks for participants are ineligible because these tasks may lead to exclusion of these vulnerable and hard-to-reach populations [55].

Researchers who want to investigate the usability of mHealth tools in populations that include little-experienced or inexperienced participants should take into account that the expectations of these participants may easily be exceeded resulting in high participant satisfaction outcomes regardless of the effectiveness and efficiency with which the tool is used. Satisfaction outcomes are influenced by the expectations that participants have before the test. It could be interesting to measure and further investigate computer anxiety and self-efficacy toward the use of the tested tool before and after usability testing to be able to put satisfaction outcomes into perspective.

Further research is necessary to gain more insight into the needs, preferences, capacities, values, and goals in relation to mHealth technology of people with low literacy skills, low educational levels, and no or little experience with using mobile technology. Insight is also needed into what effects meeting these user requirements will have on actual future use of these tools by these specific populations.

## Appendix

Multimedia Appendix 1 An overview of the screenshots of the Dutch Talking Touch Screen Questionnaire.

## Abbreviations

DTTSQ: Dutch Talking Touch Screen Questionnaire
eHealth: electronic health
ICT: information and communication technology
mHealth: mobile health
TSTI: Three-Step Test-Interview
